# Current Trends in the Application of Nanomaterials for the Removal of Pollutants from Industrial Wastewater Treatment—A Review

**DOI:** 10.3390/molecules26092799

**Published:** 2021-05-10

**Authors:** Geetha Palani, A. Arputhalatha, Karthik Kannan, Sivarama Krishna Lakkaboyana, Marlia M. Hanafiah, Vinay Kumar, Ravi Kumar Marella

**Affiliations:** 1 Physics Research Centre, Dhanalakshmi College of Engineering, Tambaram 601301, Chennai, India; kesangee@gmail.com; 2 Department of Physics, Arizona State University, Tempe, AZ 85287, USA; llathu@yahoo.com; 3 School of Advanced Materials and Engineering, Kumoh National Institute of Technology, 61 Daehak-ro, Gum-si, Gyeongbuk 39177, Korea; karthikkannanphotochem@gmail.com; 4 School of Ocean Engineering, Universiti Malaysia Terengganu, Kuala Nerus 21030, Terengganu DarulIman, Malaysia; 5 Department of Earth Sciences and Environment, Faculty of Science and Technology, Universiti Kebangsaan Malaysia, Bangi 43600, Selangor, Malaysia; mhmarlia@ukm.edu.my; 6 Centre for Tropical Climate Change System, Institute of Climate Change, Universiti Kebangsaan Malaysia, Bangi 43600, Selangor, Malaysia; 7 Department of Biotechnology, Indian Institute of Technology Roorkee, Roorkee 247667, Uttarakhand, India; vkmjnu@gmail.com; 8 Department of Chemistry (H & S), PACE Institute of Technology & Sciences, Ongole 523001, Prakasam, India; ravikumarmarella@gmail.com

**Keywords:** nanomaterials, rare earth metals, wastewater treatment, pollutants

## Abstract

In the recent decades, development of new and innovative technology resulted in a very high amount of effluents. Industrial wastewaters originating from various industries contribute as a major source of water pollution. The pollutants in the wastewater include organic and inorganic pollutants, heavy metals, and non-disintegrating materials. This pollutant poses a severe threat to the environment. Therefore, novel and innovative methods and technologies need to adapt for their removal. Recent years saw nanomaterials as a potential candidate for pollutants removal. Nowadays, a range of cost-effective nanomaterials are available with unique properties. In this context, nano-absorbents are excellent materials. Heavy metal contamination is widespread in underground and surface waters. Recently, various studies focused on the removal of heavy metals. The presented review article here focused on removal of contaminants originated from industrial wastewater utilizing nanomaterials.

## 1. Introduction

One of the common natural resources in the world is water, which is indispensable for the endurance of every human and human development. With the rapid increase of urbanization and industrialization, water usage is expanding quickly, and the problem of water shortage has become imperative to solve for developing economies. A huge quantity of polluted wastewater is released from various industries, which includes the manufacturing of batteries, mining, toxins, and electroplating. Wastewater toxins cause numerous antagonistic impacts on living creatures and to the surrounding environment [1,2]. It ended up being a more productive and more affordable tool for treating industrial wastewater. Contaminant types present in the industrial wastewater rely upon the production process [3]. Toxins of industrial wastewater usually include large constituents of organic compounds, increased pH level, harmful substantial metals, huge saltiness, and increased turbidity from the presence of impurities of inorganic compounds. Adsorption, flotation, chemical precipitation, membrane filtration, flocculation, and coagulation are included in the industrial wastewater treatment [4,5].These wastewater treatments are sometimes insufficient in eliminating explicit impurities, for example, harmful heavy metals, oil, and microorganisms.

**Figure 1:**
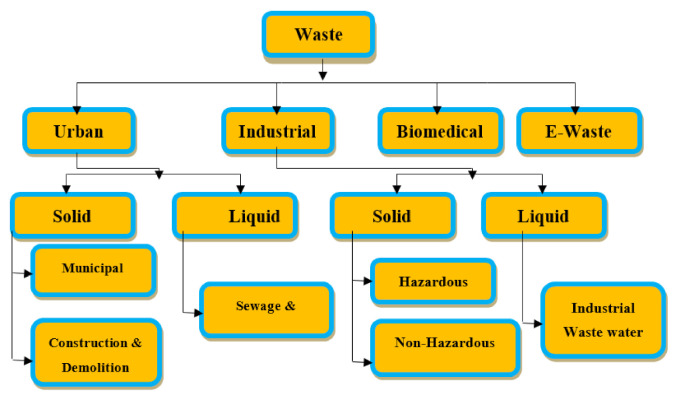
Anthropogenic wastes classification, (Reprinted with permission from [8]).

**Figure 2:**
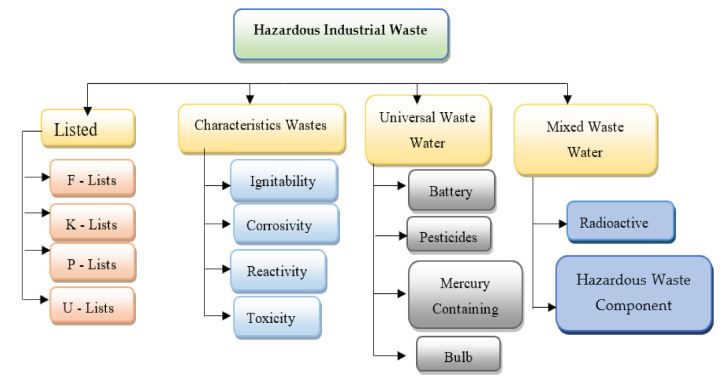
Industrial hazardous wastes general classification (reprinted with permission from [8]). F-list (wastes from common manufacturing and industrial processes), K-list (wastes from specific industries), P-list, and U-list (wastes from commercial chemical products).

Wastewater is generated from various sources such as residential areas, commercial areas, industrial properties, and agriculture lands. Composition of wastewater differs extensively, and it is majorly dependent on the source from which it is generated. Common constituents of wastewater are inorganic substances such as solutes, heavy metals, metal ions, ammonia, and gases, complex organic compounds such as excreta, plants material, food, protein, natural organic matter, and nitrate, and other pollutants present in surface water, ground water, and/or industrial water. Typically, industrial waste can be divided into two categories, hazardous and nonhazardous. Nonhazardous industrial wastes do not cause environmental and health hazards and are produced from cardboard, plastic, iron, glass, stone, and organic waste. In contrast, hazardous wastes are industrial waste that can be harmful to public health or the environment, such as flammable, biodegradable, and hazardous materials [6]. Industrial waste is classified as wastewater, solid waste, or air leaks. There is some overlap in the physical properties of the substances present in these three categories, as wastewater can contain suspended solids and suspended liquids, and precipitation of solid waste can include gas, liquid, and some liquids. Particles and air exposures may consist of a fluid that emits air fluid and a substance known as particle emission [7]. Industrial waste, which has a significant concentration of non-recyclable or recyclable metals, is usually a good candidate for landfill, which is the dumping of waste into the ground area. Figure 1 and Figure 2 shows wastewater and industrial wastewater general classifications.

When left untreated, these constituents may pose a threat to living beings and the environment, which makes it crucial to treat wastewater before disposal. Various physical, chemical, and biological treatment processes are used for wastewater treatment. Until now, a variety of research on nanomaterials was done to research heavy metal water treatments to find their applications, and they show incredible potential as an irreplaceable option to adsorb heavy metals from wastewater [8]. For the removal of heavy metals from polluted wastewater, these properties are very useful. According to the type of nanomaterial, wastewater treatment is classified into three fundamental groups [9]: nano-adsorbents, nanomembranes, and nano-catalysts. Some of the common sources of wastewater types are below [10]:

- Wastewater from municipal/domestic: discharged wastewater from habitations, foundations such as schools and medical clinics, and business offices, for example, shopping centers, restaurants, and so forth.
- Wastewater from industries: industrial processes removing wastewater, for example, drug, textile, and poultry industries.
- Infiltration/inflow: water that, in the long run, enters the sewer from establishment channels, pipes leaking, submerged manholes, groundwater invasion, etc.
- Storm water: rainfall runoff and snowmelt

Industrial wastewater treatment needs different successive complicated studies to fulfill the standard of water reusability. In looking at nanomaterials in waste streams and possibilities for recovery, one of the first items that need to be understood is the amount of industrial nanomaterial being produced and how much is then discharged, as shown in Table 1 [11]. The amount discharged to waste was based on presumptive material flow and then back calculated by knowing the reported amount produced rather than the actual measurement of the true nanomaterials concentration in a waste stream. For example, iron, zinc, and copper oxides would likely not last very long in the nanoparticle form, as they are more soluble. On the other hand, TiO_2_ is one of the least soluble among nanomaterials and would likely stay in the nanoparticle form for much longer. SiO_2_ would also be very similar to TiO_2_ with a low solubility.

One of the most significant advancements in the 21st century was nanotechnology. Nanomaterials criteria include well-organized structure, filtration competence, small in size, and high surface to volume ratio. Some special properties of nanomaterials under the nanoscale are effects on the surface region, large quantum tunnel effects, small size effects, and quantum effects. These properties add to their adsorption capacity and reactivity, which are unprecedented and are great for heavy metal ions removal [12,13]. Industrial pollution continues to be a major factor in worsening the environment around us, the water we use, the air we breathe, and the land we live in. The growing power of industrialization not only consumes large agricultural land but at the same time causes environmental degradation as well as land degradation.

For raw materials management and manufacturing related to human activities, materials are driven from the liquids of industrial wastewaters (IWW) [14,15]. IWW acts as one of the pollutants of environmental water. From a recent survey, a huge amount of industrial wastewater is mixed into lakes, beaches, and streams. In the end, this produces contamination complications and entanglements in the surrounding water and leads to the eco-system returning as a negative output that negatively impacts human life. Industrial wastewater results in human population spill, and the climate ends up being awful in many scenarios. Huge quantities of these kinds of wastewaters are naturally incredibly solid, highly inorganic, effectively biodegradable, or inhibitory potential. In regard to these qualities, total dissolved solids (TSS), biological oxygen demand (BOD), and chemical oxygen demand (COD) might be high [16]. Industrial wastewater comprises wastewater from each sector of industry that produces its own exact blend of impurities.

As with the industrial wastewater’s shifting character, industrial wastewater processing must be arranged explicitly for the specific sort of produced liquid. The metal industries discharge heavy metals and some of their compounds; also, the electroplating industry is a critical cause of contamination [17]. Higher amounts of Ag compounds are produced by photograph handling workshops, and, at the same time, printing plants release inks and dyes. The chlorine substances generally rely upon mash and paper industries; generally, they contain chloride compounds and dioxins. A very large quantity of phenols and oils are released by the petrochemical industry. Food handling effluents of the plant are loaded with organic and solid issues. Commonly, industrial wastewater is categorized into two classes: (1) organic industrial wastewater and (2) inorganic industrial wastewater. The main compound, inorganic industrial wastewater, exists in steel and coal industries, non-metallic minerals industry, metals manufacturing surface processing, and in commercial adventures [18]. Generally, wastewater, solid substances, and oils are delivered, and they contain incredibly poisonous solutes. This type may impact gas washing wastewater or cyanide blast-furnace industrial wastewater of metal processing with alkaline or acids solutions in which wastewater exudes from the gas refinement of Al work, which contains fluoride.

According to local regulations, non-metallic minerals exist in tiny and normal sizes alongside metal handling plants and are situated so they may discharge their wastewater into municipal wastewater systems; their effluents should be treated before liberation. The contaminants that come from the chemical compounds/industries which act on a large-scale by organic industrial wastewater mostly exploit substances for chemical responses [19]. The fluids incorporate substances of organics with variable properties and beginnings. The below-listed plants and industries primarily produce organic industrial wastewaters (OIW) [20]:factories manufacturing pharmaceuticals, beauty products, synthetic detergents, herbicides, and pesticides, leather and tanneries factories, textile factories, paper and cellulose manufacturing plants, factories related to oil-refining, and metal processing industry.

Now, the steel industry is viewed as a fundamental and crucial industry. Steel industries produce wastewater in huge quantities that contain many disintegrated chemicals in the sludge and undisclosed substances [20]. Producing iron out of its metals involves intensely decreased responses in impact heaters. Necessary cooling waters such as cyanide and ammonium hydroxides are soiled. Acidified rinse waters are present in wastewaters and waste corrosive blends. Regardless of the large number of plants working in acid plants recovery, where the mineral acid is reduced from the salts of iron, a huge amount of acid ferrous sulfate/chloride remains undisposed [21]. The paper production and wood-pulping products distribute polluters when natural fluids are delivered into emanating waters [22]. These liquids subsequently have extraordinary mutagenic effects that cause physiological weakness and damage aquatic organisms. The textile industry altogether additionally adds to water contamination in changing produced and natural threads into fabrics and different products. While fabricating many of the materials, wet chemical techniques are compulsory to legitimately sanitize, color, plan, or finalize the item [23]. This represents wastewater development, which usually contains loads to eliminate pollution from the crude materials themselves, yet excess reagent compounds are utilized in preparing such processes. Essential polluters in textile wastewaters are exceptionally chemical oxygen, heat, suspended solids, corrosiveness, and some dissolvable substances [24].

### Industrial Wastewater Treatment (IWT) Processes

IWT is commonly arranged as physical, chemical, and organic processes. The usually embraced advances might be divided into [25]: (i) pre-treatment, (ii) primary; (iii) secondary and tertiary; (iv) refinement; and (v) purification. Usually, a basic level of treatment is based on size divisions utilizing physical methods, for example, filtration/sedimentation, for fundamental cleaning. More than 99% of removal can be achieved by tertiary treatment, which involves the final polishing of the effluent by toxic removal of pollutants to certain levels [26,27,28]. Wastewater produced by the primary process is not reasonable for release or reuse. The primary goal is to deliver the water suitable for optional and tertiary separations. A noticeable illustration of this is pH clarification/modification before membrane adsorption or separation or ion exchange. Separation processes, which are ordinarily viewed as basic treatments, include separation based on size, including the actual driving force that affects separation. Screening, cyclone separations, sedimentation, precipitations, thickening, centrifugation, and filtration are included in primary methods. Among the main homogenization/leveling, pre-treatments are meant to balance, and wastewater homogenization from inlets, particularly where industrial production creation occurs, is irregular and variable for consumption toxins [28].

**Figure 3:**
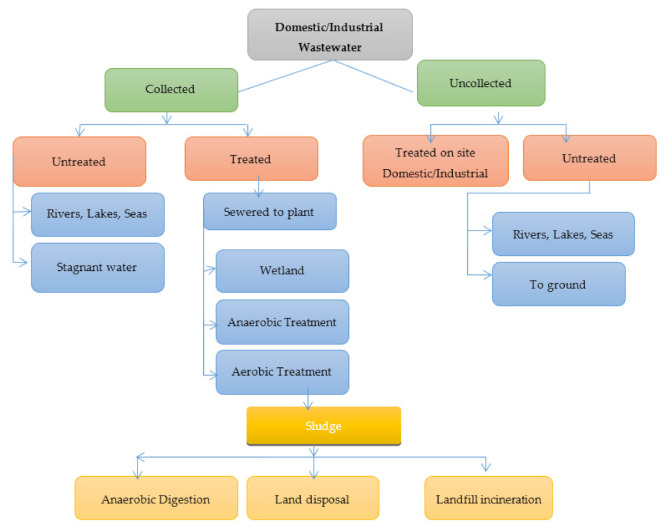
Flow chart showing steps in wastewater treatment processes.

Further developed processes of separation are utilized with immense changes in equipment and process nature in secondary and tertiary treatment stages. The separation process generally incorporates evaporation, absorption, distillation, extraction, ion exchange, biological processes, adsorption, crystallization, cavitations, and separation of membranes [29]. The process of separations involved here can be arranged depending on driving forces, such as thermal driving force, and pressure-driven processes, such as membrane separation-microfiltration (MF), ultra filtration (UF), nano-filtration (NF), and electrical forces, e.g., electro-dialysis or RO. Physicochemical methods are the main processes of separation that assume an imperative function in the field of wastewater treatment. This main class incorporates a huge assortment of processes, such as flocculation/coagulation, cavitations, oxidations, separation reactions, and extractions [30]. The process of separation includes ion exchange, and adsorption also comes under physicochemical methods of treatment for utilizing either electrostatic attraction or surface forces. Ion exchange, coagulation, and adsorption are methods of membranes separation that belong to charge-based separations, where the removal process is generally contaminated under charge neutralization and is applicable explicitly for the removal of charged bodies/ions from the solution. Based on the idea of the profluent, at least one process of separation is involved in meeting the end goals of water reuse/discharge [31]. Figure 3 shows the steps in wastewater treatment processes as a flow chart.

## 2. Removal of Dyes

From industries, waters dyes and pesticides are being released frequently, and their findings, particularly at low concentrations, need the development of complex advances, for example, separation or filtration of compound combinations combined with detection utilizing multi-technique methods [32,33]. These estimations are subsequently tedious due to the many middle-level handling steps associated with preparing the sample. Utilization of silicon-graphene (sg) nanoporous composites takes into consideration an extreme cut of all these methods, as the compounds can pre-concentrate the analyte into the porous structure and widen the analyte signal if a proper method is utilized [34]. Table 2 shows the application and the harmfulness of various dyes [35,36].

By using Raman spectroscopy [28], for example, steps in sampling the water can be removed. The specific hierarchal porous silicon graphene composites design and the thick coating formed shape permit a “lab-on-a-chip” device to be combined with the Raman scattering method. Samples of water can be straightforwardly stored over the silicon graphene coatings, and toxins can be identified with the utilization of Raman spectroscopy. Raman signal enhancement identified with the analysis can be achieved through pure graphene-enhanced Raman scattering effect (GERS) or a combination of the surface-enhanced Raman scattering (SERS), e.g., graphene-mediated, surface-enhanced Raman scattering (G-SERS) given by plasmonic nanoparticles [37,38]. The affectability of such devices can be obtained by molecular imprinting methods. This substance approach permits framing exceptionally tiny cavities in the porous silica structures with sub-atomic recognition capabilities. Using molecular templating techniques, silicon graphene is able to form thick films that can be utilized for detecting dyes that mostly show the largest analytical enhancement factor of 14.64 for Rhodamine 6G dyes for a concentration of 10^−3^ M [39,40]. Additionally, the same team prepared porous SG templated films for the detection of paraoxon, an organophosphate pesticide, with the concentration of 10^−5^ M. Aside from identification, composites of silicon graphene additionally react as sorbents for the equivalent molecules of the analyte. Adsorption of organ phosphorus pesticides (OPPs) on silicon graphene composites was researched [41].

The adsorption was helped because of the substance co-operations between the composite and the functional groups; moreover, the strong π bonding between composites and the phenyl ring preferred the adsorption. The performance of adsorption of silicon graphene compounds was explored for various pesticides, and the capacity of removal varied. As referred to recently, for this situation, the expansion of magnetic nanoparticles to silicon graphene composites assumes a significant part in facilitating the recovery of the composites. Additionally, with an extra modification in the hydrophobic surface, the adsorption sites of pesticides expand [42]. Dye adsorption onto silicon graphene composites is likewise preferred by a mixture of physicochemical adsorption routes that rely upon the sorbent physicochemical properties.

Silicon graphene nanocomposites of the multifunctional compound were prepared by Kubo et al., inducing a super paramagnetic nanoparticles material to the mesoporous silica embedding graphene oxide. The nanoparticle functionalization permitted a simple recovery of the composites by an outer magnet. Fe_3_O_4_ addition, nonetheless, caused sharp diminishing of the area of the surface by 72% and ~15% of pore sizes, which decreased from 305 mg/g to 125 mg/g in regard to methylene blue (MB) removal capacity [43,44]. Considering this, amino group surface functionalization of the multifunctional silicon graphene nanocomposites might help increase the interactions for the pollutant removal.

The sorbent physical properties, e.g., pore size, surface area, and shape, give more sites for the dyes to diffuse and secure through the pores. The performance of adsorption is reliant additionally on the physicochemical properties of the dyes and the environmental media [28,45], such as different structure charges and dyes, which show unmistakable sorption conduct in various pH conditions.

## 3. Heavy Metals Removal

**Figure 4:**
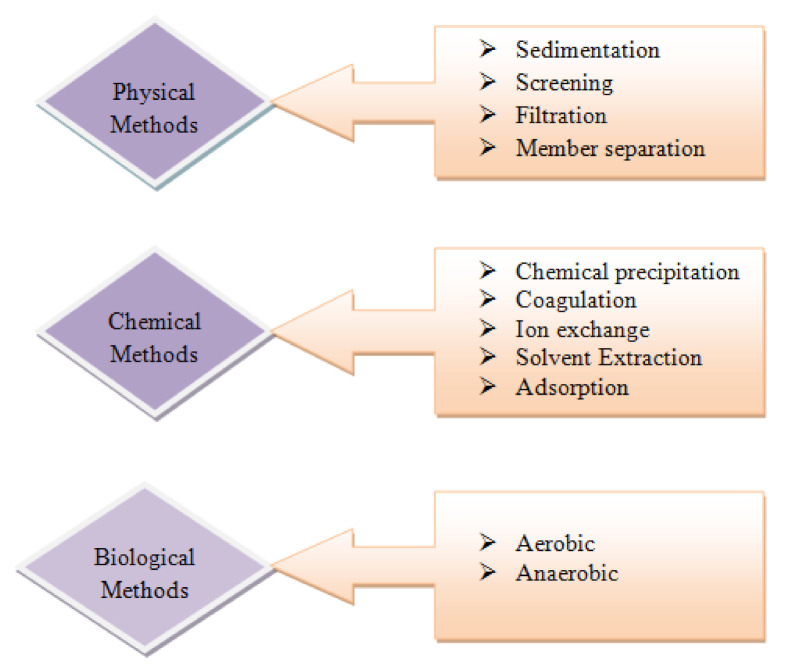
Conventional technologies for heavy metal removal, reprinted with permission from [52].

Heavy metals are those metals which have very high density and are highly poisonous, even at low concentrations. These heavy metals include mercury (Hg), cadmium (Cd), arsenic(As), chromium (Cr), thallium (Tl), zinc (Zn), nickel (Ni), copper (Cu), and lead (Pb). Municipal wastewater, industrial wastewaters, landfill leaches, mining wastes, and urban runoff are the main sources of contamination [46,47]. Industries are the major source of heavy metals in wastewater. The electroplating industry’s wastewater contains a great number of metals such as Cd, Zn, Pb, Cr, Ni, C, Ag, Pd, and titanium. The wood processing industry is also a major source of metal in wastewater. Paint and enameling industries also release their effluents containing nickel into water bodies [48]. PCB manufacturing industries are also a very significant source of producing metal waste [49]. Nanomaterials of metal compounds displayed preference for heavy metals removal over activated carbon, e.g., titanium dioxide nanoparticles in arsenic adsorption and nanosized magnetite. The photo catalyst usage of, for example, nanoparticles titanium dioxide, was explored in detail to decrease the toxic metal ions in water. In a survey, titanium dioxide nanocrystalline showed adequacy in eliminating various types of As, and it was demonstrated to be the most viable photo catalyst aside from industrially accessible nanoparticles of titanium dioxide, which showed almost extreme efficiency of arsenic removal at a relatively neutral pH value [50]. A titanium dioxide nanocomposite and nanoparticles of titanium dioxide added on a graphene sheet was additionally utilized to decrease chromium VI to chromium III in daylight. Comparatively, chromium treatment was completed by utilizing nanoparticles of palladium in another survey; conventional technologies for heavy metal removal are shown in Figure 4. The removing capacity of arsenic (heavy metal) was likewise tested by utilizing Fe_2_O_3_ and Fe_3_O_4_ as adsorbents by most of the analysts. Removal of As was also additionally explored by utilizing a high, particular surface area of iron oxide nanocrystals [51].

**Figure 5:**
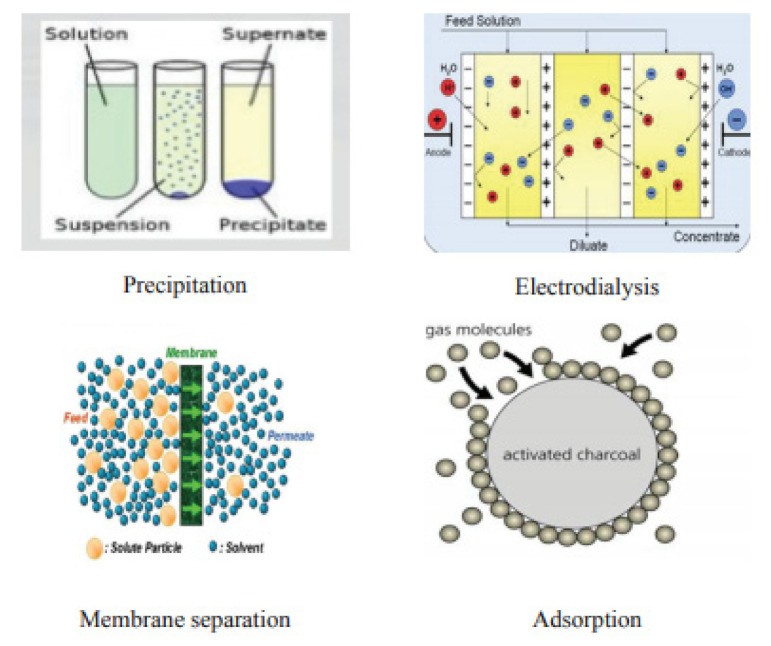
Conventional methods for removal of metal, reprinted with permission from [52].

Recently, this process was recognized widely for removal of heavy metals from wastewater. Many cheap adsorbents were developed recently. These adsorbents are widely used for treatment of wastewater containing heavy metals [52]. These adsorbents are derived from the waste products generated from industrial activities, waste generated from agriculture, and natural materials [53]. Adsorption can be defined as a mass transfer process which transfers the substance from the liquid phase to the surface of a solid and becomes bound by physical and chemical interactions. Figure 5, illustrates some conventional methods for removal of metal. It is a three-step treatment process: (1) the pollutant is transferred to the sorbent surface from bulk solution, (2) adsorption occurs on the particle surface, (3) transportation occurs within the sorbent particle. These techniques are very cost effective: adsorption on modified natural materials, adsorption on industrial by-products, bio-sorption [54].

## 4. Removal of Pesticides

Low-cost adsorbents development for pesticide maintenance is a significant part of environmental sciences research. Wastes from industries such as carbon slurry, fly ash, and sludge are delegated as easy materials due to their minimal price, and local accessibility pesticides removal can be utilized as adsorbents. Fly ash, lignite, coal-fired, thermal, power station solid waste, is an easy adsorbent that demonstrated huge adsorption limit with regard to organic contaminations [55]. Some researchers explained the sorption of pesticides and fly ash capability and suggested its utilization for pesticides removal from wastewater samples and water. Coal fly ash altogether has a high maintenance limit with regard to metolachlor, atrazine, and metribuzin.

When compared with metribuzin and metolachlor, atrazine was the most sorbed. The fly ash sorption herbicide efficiency relied upon the herbicide concentration in the mixes, and the highest removal of herbicide was seen at lower concentrations. These are common experiences in samples of water. At some point, when the water enters, pollution of biodegradables goes through concentrated processes of biological disintegration. Pollution of bio-resistant items fundamentally presents, to a lesser extent, an issue on the off chance that they are inactive biologically or are inorganic, inert, material framing sludges that are environmentally appropriate for benthos production and biological processes improvement. Inorganic poisonous contamination [56] is caused by heavy metals, organic pesticides, and so on, where nature speaks to the most unsafe kind of bio-resistant pollution.

The health risk from unsafe and hazardous chemical substances present in drinking water is regularly characterized as the likelihood that an unfriendly impact on health will be caused by substance exposure. The microelements interact and transport particularly heavy metals; streams courses indicate the most complex aquatic systems. In the agricultural region, water pollution is mainly caused by fertilizers, pesticides, and waste from poultry processing plants, drainage from livestock farms, and so on. Particularly, pollution made by pesticides on the row by heavy metals from fertilizers and nitrates contamination is very dangerous. Water mineralization indicates groundwater contamination of soil improvement on territories. Large mineral exploitation stores frequently prompt water quality disintegration on the more extensive area around strip mines and mines [57]. A great deal of water is commonly cleared during the depositing of drainage, and it upsets the groundwater regime and disturbs the hydro-chemical equilibrium.

## 5. Key Parameters for the Pollutant Removal

Commonly, contaminations can exist in viscous crude oil forms that are mostly either miscible or immiscible in water or as heavy metals, dyes, and pesticides of dispersible molecules. From the accessible information, there is more to viscous oil capturing than the actual process, and it intensely relies upon the huge composites’ porosity and density for dissemination of fluid density via pores. Fewer viscosity solvents stream into the pores easily, but highly viscous liquids are used to diffuse slowly. The multifunctional composites design can be heated up through various mechanisms by using Joule effect, which permits one to reduce the oil viscosity, allowing faster diffusion of oil into the pores and promoting quicker removal. On the account of water-dispersible contaminations, the elimination depends on electrostatic interactions and chemicals with the sorbent. These may be improved by expanding the number of dynamic sites such as surface area and charge by chemical functionalization.

Silicon graphene multifunctional composites can be surface-functionalized to enhance the modifications with the analyte. The technique of sub-atomic is a wiser approach to silicon graphene composites with improved performance [58,59]. The molecular cavities focus on molecules and improve the detecting capabilities of dyes. The molecular imprinting is likewise useful for designing nanocomposites of silicon graphene for removing heavy metals. Moreover, conditions of the environment play a critical function in the pollutant’s adsorption.

In general, the process of sorption relies on the physical state and the physicochemical properties of the composite and the pollutant chemical composition in the water. The properties evaluation versus the performance of the material is significant due to the absence of broad availability of characterization data and associated sorption capacities, such as specific surface areas [60,61]. Regardless of these obstructions, we distinguished the patterns and were effective in building up connections that helped increase the performance of the composites.

## 6. Nanomaterials Reuse and Retaining

Nano-adsorbent reuse and materials recovery from an aqueous solution are profoundly hard and may cause problems to the environment; as the compounds are adsorbed, pre-treatment samples must be created, and the process of separation technologies must ensue [62]. As another option, ballistic electrons discharged from the nanostructured adsorbent material and the nonporous carbonaceous utilizing microwave irradiation may likewise annihilate the adsorbed compound. Nanomaterial holding and reutilization by membrane filtration are enabled device designs of nanotechnology, key features that allow persistent chemical usage because of the expense and general health concerns. Besides, membranes of ceramics are beneficial compared to polymeric membranes in photo catalytic applications, as they exceptionally oppose chemical oxidants and ultraviolet.

Nanomaterials can be controlled on different membranes and resins, thus separation can be removed further. Vast studies are needed to propel easy, budget free techniques for nanomaterials immobilization without influencing their performances. In any case, the liberation potential is required to be generally reliant on the separation process and the technique of immobilization employed. For nanomaterials, which liberate metal ions, their disintegration must be restrained carefully [63,64]. The nanomaterial liberation detection is a significant, specialized obstacle for hazardous assessment and remains challenging, retaining particular nanomaterials reutilization to correct the cost process. For a nanomaterial applicable to treat wastewater, there are two investigative requirements. Future research with good, consistent conditions is needed to utilize various nanomaterials efficiencies.

## 7. Environmental Significance and Future Application

As of now, there is no doubt of the nanomaterials usage efficiency in industrial wastewater treatment, however, nanomaterials impose a huge number of genuine cons because, during treatment processes, they may discharge into the surrounding environment, and they can withstand serious risks for a long time. In such a manner, there is a requirement for more studies and surveys to decrease the toxicity in the environment [40,65]. Among different metal oxide nanoparticles, titanium dioxide, for instance, is generally utilized and have some significant constraints and some toxic effects towards human health and the environment, which causes difficulty in creating a sustainable environmental pollutants removal system. In such a manner, initiatives for new findings and research should be investigated to overcome these difficulties, and several scientists and analysts are continuously attempting to defeat these obstructions [66]. A few researchers just determined a better approach to diminish the band gap to utilize solar energy proficiently [67]. More research is needed to build up the practical techniques for the synthesis of nanomaterials and to determine effective areas of application in discovering the material proficiency. Table 3 summarizes the advantages and the disadvantages of the different physicochemical technologies for treatment of rare-earth elements in water and wastewater.

The market requirement is to restrict the costs of the procedure concerning the environmentally friendly nature of the referenced nanomaterials. Critical accentuation for this utilization should be given to green technology by material by-products of agricultural wastes. Numerous works in this area are needed in order to improve. The main concentration of the existing research works is that their utilization is yet to occur in the research [68,69]. Only a restricted amount of studies for brief market analyses and economic aspects is accessible. The principle market objective for the future is to improve the process of treatment on an industrial scale, which requires a significant monetary and technological method. In such a way, colleges and research labs can assume an effective part through a more official way to deal with the transfer of technology and copyright protection [70,71,72].

## 8. Conclusions

Water safety is of critical importance among major areas in the universe because of population increase, droughts drawn out, environmental changes, etc. From the literature review, wastewater or water treatment utilizing nanomaterials is becoming a prominent field in research. When compared with other planets, water makes our planet better. However, the overall pure water accessibility is part of what is causing current and unsurprising demands for water. The resources of drinking water are not satisfying domestic developmental, fundamental, or basic needs in many regions of the world. In particular regions, there is a lack of pure water to satisfy the fundamental requirement for sanitation, and human water needs are positively a breaking point in regard to the well-being of humans and for other creatures in the world. Academicians, research institutes, research fellows, and young scientists must discover a new way to eliminate these constraints. This universe is confronting many difficulties in doing this, particularly given a fluctuating and environmental future; a rise in population is driving community enlargement, globalization, and urbanization. How to preeminently defeat these difficulties requires investigation in every aspect of water management.

Nanomaterial treatment for water pollutants is becoming a trend, and it is drastically improving in this advanced time because of entirely awful states of water and freshwater demand in the entire universe. A significant requirement for progressive innovation for water treatment draws near, explicitly to affirm good quality drinking water and to eliminate micro and macro contaminations and toxins. We must improve developments of industrial production through deftly replaceable approaches for water treatment. Nanotechnology has manifested incredible accomplishments for water decontamination, controlling difficulties and making some progress for the future. Approaches of nanomaterials such as nanostructured catalytic membranes, nanosorbents, and so on are extremely productive, require less time, are eco-friendly techniques, and require less energy, however, every one of these techniques is inexpensive, and they are not utilized at this point for the industrial purpose of wastewater purification at an enormous scope.

Due to the high reaction rate, nanomaterials show high efficiency. In any case, there are a few shortcomings that should be avoided. There is no digital, computerized monitoring methods that offer predictable measurement on real-life facts regarding nanoparticles prevalence, which are available in limited quantities in water. Besides, to decrease hazards to health, international research universities and research institutes ought to plan legitimate terms and conditions to solve these circumstances. Moreover, the nanoengineered mechanical restriction for approaching water is that it is inconsistently adaptable to mass growths, and in today’s scenario, few cases exist that are not obtrusive with moderate treatment approaches. Moreover, there are incredible requirements to incorporate some nanomaterials modifications, which include having large productivity and being cost effective, simple to deal with, and environmentally friendly. It is likewise important for wastewater treatment to grasp the economic difficulties and the commercialization of these innovations. Various uses of nanomaterial can give a gigantic proposal to flexible drinking water to the entire universe.

## Figures and Tables

**Table 1:** Nanomaterials and discharges (reprinted with permission from [11]).

Si. No	Nano Material	Discharge
1	Silica (SiO_2_)	95,000
2	Titania (TiO_2_)	88,000
3	Alumina (Al_2_O_3_)	34,900
4	Zinc oxides	34,100
5	Nano-clays	10,400
6	Cu and Cu oxides	497
7	Ag	424

**Table 2:** Application and harmfulness of various dyes, reprinted with permission from [35,36].

Dyes	Example	Advantages	Toxicity
Acid	Methyl orange, Sunset yellow	Wool, paper, leather, silk	Carcinogenic
Cationic	Rhodamine 6g, Methylene blue	Paper modified polyesters	Carcinogenic
Direct	Congo red, Direct red 23	Cotton, paper, leather	Bladder cancer
Disperse	Disperse red, Disperse orange 3	Nylon, acrylic fibers	Skin allergenic
Reactive	Reactive red 198 Reactive red 120	Nylon, wool, cotton	Dermatitis, allergic conjunctivitis
Vat	Vat orange 28, Vat orange 50	Cellulosic fibers	

**Table 3:** Summary of the advantages and the disadvantages of the different physicochemical technologies for treatment of rare earth elements in water and wastewater [72].

Si.No.	Removal Technologies	Advantages	Disadvantages
1.	Chemical precipitation	Simple and safe operation Low capital cost Most metals can be removed	The increased operational ease disposal of sludge Slow metal precipitation kinetics
2.	Electrocoagulation	The high particulate removal efficiency Relatively low cost Compact treatment facility	Sacrificial anodes need to be replaced periodically Sludge production High operating cost
3.	Flotation	Low sludge generation Low cost and low energy requirements	To develop the removal efficiency treatments are required
4.	Ion exchange	Selection of metals High regeneration of materials Less time consuming High removal capacity	for metal removal not all resin ion exchange is suitable Regeneration creates a sludge disposal problem High Capital cost
5.	Biosorption	Use of inexpensive biosorbents Regeneration of biosorbent and low operating cost Selectivity and efficiency High metal binding capacity	The potential for biological process improvement is limited. Separation of biosorbents is difficult after adsorption Early saturation
6.	Ionic imprinted polymer	Stable and easy to prepare Inexpensive Metal selective	Polydispersity nature of the recognition sites Difficult to characterize

## Data Availability

Not applicable.
